# Ischemia prediction score (IsPS) in patients with strangulated small bowel obstruction: a retrospective cohort study

**DOI:** 10.1186/s12876-023-02761-z

**Published:** 2023-04-24

**Authors:** Shuhei Murao, Shiki Fujino, Katsuki Danno, Takashi Takeda, Kei Yamamoto, Masaya Higashiguchi, Kozo Noguchi, Takafumi Hirao, Yoshio Oka

**Affiliations:** Departement of Surgery, Minoh City Hospital, 5-7-1 Kayano, Minoh, Osaka 562-0014 Japan

**Keywords:** Strangulated small bowel obstruction, Intestinal ischemia, Emergency surgery, Computed tomography, Prediction model

## Abstract

**Backgrounds:**

Intestinal ischemia of strangulated small bowel obstruction (SSBO) requires prompt identification and early intervention. This study aimed to evaluate the risk factors and develop a prediction model of intestinal ischemia requiring bowel resection in SSBO.

**Methods:**

This was a single-center, retrospective cohort study of consecutive patients underwent emergency surgery for SSBO from April 2007 to December 2021. Univariate analysis was performed to identify the risk factors for bowel resection in these patients. Two clinical scores (with contrasted computed tomography [CT] and without contrasted CT) were developed to predict intestinal ischemia. The scores were validated in an independent cohort.

**Results:**

A total of 127 patients were included, 100 in the development cohort (DC) and 27 in the validation cohort (VC). Univariate analysis showed that high white blood cell count (WBC), low base excess (BE), ascites and reduced bowel enhancement were significantly associated with bowel resection. The ischemia prediction score (IsPS) comprised 1 point each for WBC ≥ 10,000/L, BE ≤ -1.0 mmol/L, ascites, and 2 points for reduced bowel enhancement. The simple IsPS (s-IsPS, without contrasted CT) of 2 or more had a sensitivity of 69.4%, specificity of 65.4%. The modified IsPS (m-IsPS, with contrasted CT) of 3 or more had a sensitivity of 86.7%, specificity of 76.0%. AUC of s-IsPS was 0.716 in DC and 0.812 in VC, and AUC of m-IsPS was 0.838 and 0.814.

**Conclusion:**

IsPS predicted possibility of ischemic intestinal resection with high accuracy and can help in the early identification of intestinal ischemia in SSBO.

**Supplementary Information:**

The online version contains supplementary material available at 10.1186/s12876-023-02761-z.

## Introduction

Strangulated small bowel obstruction (SSBO) is one of the leading causes of acute abdomen that requires an emergency operation and accounts for 20% of emergency surgical procedures [1]. When intestinal ischemia or necrosis occurs in SSBO, delayed diagnosis and intervention can result in a higher incidence of mortality, ranging from 8 to 25% [2]. However, the evaluation of intestinal ischemia is not well standardized, and most non-specialized clinicians are often unable to distinguish between a critical SSBO requiring bowel resection and others.

Several studies have reported the various clinical, laboratory and radiological risk factors for intestinal ischemia in SSBO patients. Clinical signs of intestinal ischemia include fever, pain duration, guarding, leucocytosis, peritoneal fluid, or reduced bowel contrast enhancement on computed tomography (CT) [3–5]. However, each clinical parameter alone was poorly predictive with a sensitivity of 48% in physical examination or a diminished specificity (50–64%) of bowel ischemia as a radiological sign [6–8]. Thus, there is no consensus on indications for surgical small bowel resection for specific SSBO patients. Moreover, in most studies, radiological signs were based on the evaluation of experienced radiologists, which is not available in some clinical settings, and thus there is a need for objective radiological assessment with widely applicable criteria.

This study evaluated the risk factors for bowel resection in SSBO patients who underwent emergency laparotomy using objective radiological criteria for assessing CT. Thereafter, we developed and validated the prediction score for the need of bowel resection in SSBO.

## Methods

### Study design

This was a single-center, retrospective cohort study conducted at a tertiary hospital in Osaka, Japan. Records of patients who underwent operation after being diagnosed with strangulated small bowel obstruction between April 2007 and December 2021 were retrospectively reviewed. Exclusion criteria were large bowel obstruction, incarcerated abdominal wall hernia, femoral hernia and patients who received conservative treatment initially. The included patients were divided into 2 cohorts according to the operation period. The development cohort (DC) included patients treated between April 2007 and December 2018, and the validation cohort (VC) included patients treated between December 2018 and December 2021. The study followed the Declaration of Helsinki and was approved by the Institutional Review Board at Minoh City Hospital (R0311B64).

### Data collection

**Fig. 1:**
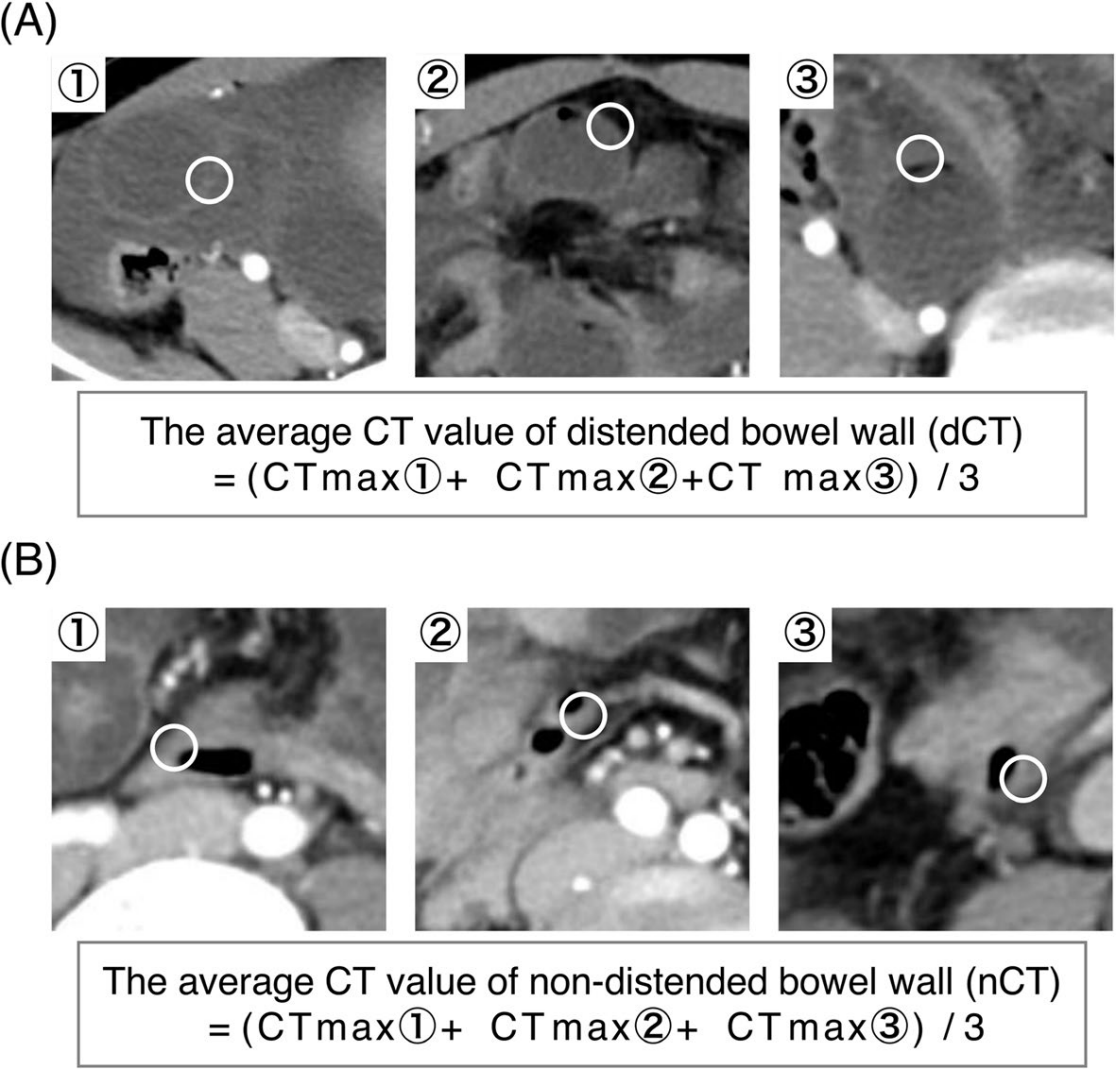
Measurement method of the CT value of the small bowel wall. 10 mm-white circles were shown as region of interest (ROI). The small-bowel wall was contained in ROI. The highest CT value of ROI was set as the CT value of the wall. Three different sites were measured. **A** Representative figure of the measurement of distended small bowel. The average value was calculated as the average CT value of distended bowel wall (dCT). **B** Representative figure of the measurement of non-distended small bowel. The average value was calculated as the average CT value of non-distended bowel wall (nCT). CT, computed tomography. ROI, region of interest

All clinical and biological data were collected during the admission and included age, sex, past-history of surgery, American Society of Anesthesiologists physical status (ASA-PS), duration of symptoms before surgery, peritoneal irritation signs (guarding, rebound) and body temperature. Blood tests included white blood cell (WBC) count, level of C-reactive protein (CRP), lactate dehydrogenase (LDH), creatine kinase (CK) and blood gas (pH, PaCO_2_ and Base Excess). CT findings included the presence of ascites, mesenteric fluid and reduced bowel wall enhancement. Reduced wall enhancement was evaluated as discussed here. The CT value was continuously measured from the inner side to the outer side of the distended small-bowel wall with a circle of 10 mm diameter as region of interest (ROI) and the highest value was set as the CT value of the wall. We measured the CT value at any three sites and the average value was calculated. The same method was carried out in non-distended bowel. To measure the CT values in the non-ischemic small bowel, the non-distended small bowel was defined as the small intestine away from the lesion as the measurement point. Thereafter, ROC curve analysis was used to explore the optimal cutoff point. Based on the present cut-off point and the previous study which also evaluated the CT value of intestinal ischemia of SSBO patients [9], reduced wall enhancement was defined as the average CT value of the distended small-bowel wall decreased by 30% compared to that of the non-distended bowel (Fig. 1, Figure S1). There were two clinical outcome categories: patients who underwent laparotomy but had no evidence of ischemia and no resection, and patients who underwent laparotomy with evidence of intestinal ischemia requiring small bowel resection. There were no cases in which intestinal resection was performed for reasons other than ischemia, and in which ischemia progressed and additional bowel resection was performed later.

### Statistical analysis and the creation of the clinical score to predict intestinal ischemia

Continuous variables were divided into clinically meaningful categories and compared using χ2 tests. Logistic regression analysis was performed to explore risk factors requiring bowel resection in patients with strangulated small bowel obstruction. Odds ratios were also evaluated and *p* values < 0.05 were considered statistically significant. The ischemia prediction scores (IsPS) were constructed on the basis of the univariate logistic regression analysis. IsPS was structured using DC and validated in VC. A receiver operating characteristic (ROC) curve was obtained and the area under the curve (AUC) was calculated to assess the discriminant ability of these scores. All statistical analyses were performed using the JMP 13.0 statistical software program (SAS Institute, Cary, NC, USA).

## Results

### Patient characteristics

**Fig. 2:**
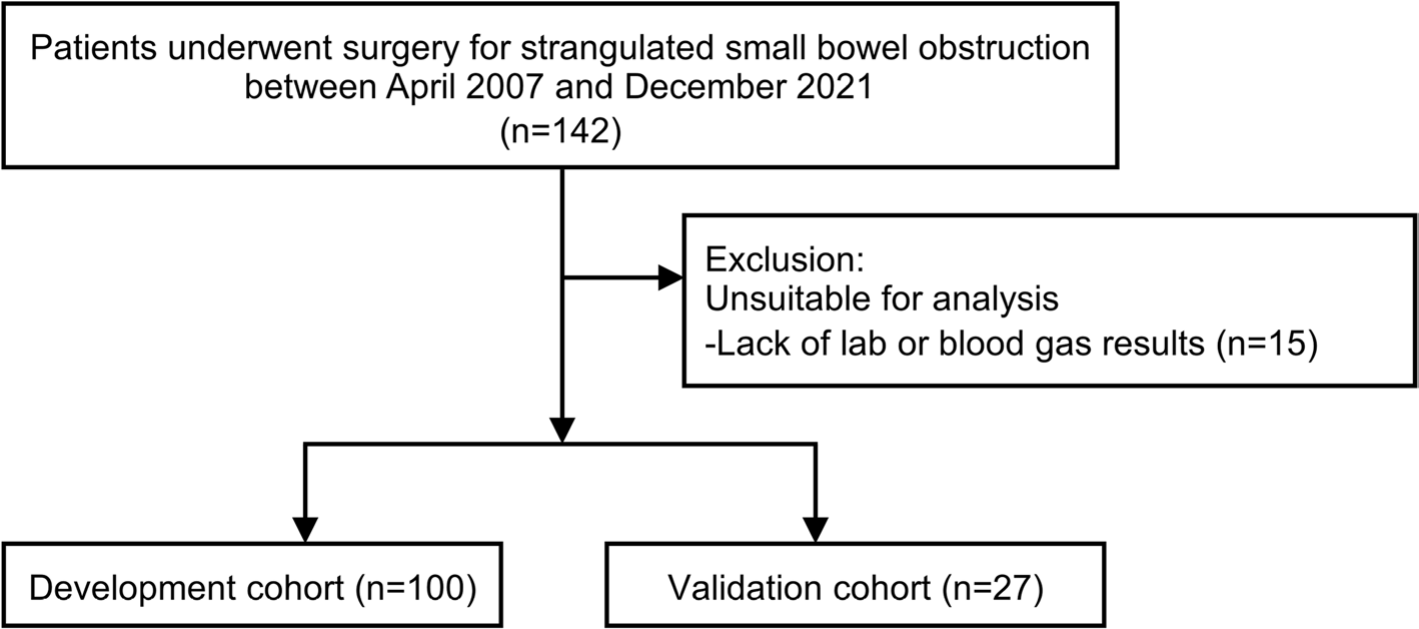
Patient flow chart

One hundred and forty-two patients who underwent emergency surgery with strangulated small bowel obstruction between April 2007 and December 2021 were included. Fifteen patients with lack of laboratory data were excluded, and 127 patients were divided into two cohorts; 100 patients in the DC and 27 patients in the VC (Fig. 2). The characteristics of all 127 study patients (DC and VC) are listed in Table 1.

**Table 1:** Patients characteristics

	**Development** **cohort (** ***n*** ** = 100)**	**Validation** **cohort (** ***n*** ** = 27)**
Background		
Age, years^a^	69 (57–82)	72 (54–85)
Sex, male/female	37 / 63	13 / 15
Past history of surgery (absent/present)	62 / 38	15 / 12
Duration of symptoms before surgery, hours ^a^	12 (8–21)	15 (8–26)
ASA-PS (1/2/3)	13 / 52 / 35	5 / 14 / 8
Physical findings		
Peritoneal irritation sign (absent/present)	39 / 61	7 / 20
Body temperature, °C^a^	36.6 (36.2—36.9)	36.9 (36.6—37.1)
Laboratory data		
WBC count, 10^3^/L^a^	9.5 (7.5—12.9)	10.0 (8.0—13.1)
CRP, mg/L^a^	0.20 (0.10—0.73)	0.18 (0.10—1.30)
CK, U/L^a^	88 (50—126)	82 (58—125)
LDH, U/L^a^	210 (181—252)	200 (176—223)
Arterial blood gas		
pH^a^	7.44 (7.41—7.47)	7.44 (7.41—7.45)
BE, mmol/L^a^	0.2 (-2.4—1.4)	0.8 (-0.9—2.0)
CT findings		
Ascites (absent/present)	75 / 25	17 / 10
Mesenteric fluid (absent/present)	73 / 27	18 / 9
Reduced bowel wall enhancement (absent/present)	40 / 44	10 / 11
Surgery		
Small bowel resection (absent/present)	36 / 64	10 / 17
Etiology of SSBO based on surgical findings		
Adhesive disease	86 (86%)	22 (81%)
Internal hernia	11 (11%)	5 (19%)
Volvulus	3 (3%)	0

*ASA-PS* American Society of Anesthesiologists physical status, *WBC* White blood cell, *CRP* C-reactive protein, *CK* Creatine kinase, *LDH* Lactate dehydrogenase, *BE* Base excess, *CT* Computed tomography

^a^Median (range)

### Univariate analysis of predictive factors of intestinal ischemia in the development cohort

In the DC, 36 patients (36%) required small bowel resection because of ischemia or necrosis (ischemia group), and 64 patients underwent surgery without the need for small bowel resection (no ischemia group). The results of univariate analysis in the DC are shown in Table 2. Higher white blood cell count (WBC ≥ 10,000/L), lower base excess (BE ≤ -1.0 mmol/L), and ascites were significant factors in the resection group.

**Table 2:** Predictive factors of intestinal ischemia in development cohort

	**No ischemia group** **(** ***n*** ** = 64)**	**Ischemia group** **(** ***n*** ** = 36)**	***P***	**OR (95% CI)**
Background				
Age years ≥ 60 years	46 (72%)	28 (78%)	0.515	
Sex, male/female	24 / 40	13 / 23	0.890	
Past history of surgery	38 (59%)	24 (67%)	0.469	
Duration of symptoms before surgery ≥ 24 h/ < 24 h	14 (22%)	9 (25%)	0.667	
ASA-PS ≥ 2	53 (82%)	34 (95%)	0.078	
Physical findings				
Peritoneal irritation sign	19 (30%)	12 (34%)	0.638	
Body temperature ≥ 37.5 °C	6 (10%)	1 (3%)	0.228	
Laboratory data				
WBC count ≥ 10,000/L	21 (33%)	21 (58%)	0.013	2.86 (1.23—6.66)
CRP ≥ 5.0 mg/L	4 (6%)	3 (8%)	0.698	
CK ≥ 200U/L	6 (10%)	3 (9%)	0.867	
LDH ≥ 300U/L	4 (6%)	5 (14%)	0.228	
Arterial blood gas				
pH ≤ 7.35	0 (0%)	1 (3%)	0.153	
BE ≤ -1.0 mmol/L	16 (25%)	17 (47%)	0.024	2.68 (1.13—6.38)
CT findings				
Ascites	43 (67%)	32 (89%)	0.012	3.91 (1.22—12.5)
Mesenteric fluid	44 (69%)	29 (81%)	0.194	
Reduced bowel wall enhancement	16 (30%)	24 (80%)	< 0.001	9.50 (3.26—27.6)

Categorical variables are presented as numbers (%)

*ASA-PS* American Society of Anesthesiologists physical status, *WBC* White blood cell, *CRP* C-reactive protein, *CK* Creatine kinase, *LDH* Lactate dehydrogenase, *BE* Base excess, *CT* Computed tomography

Eighty-four people underwent contrast-enhanced CT, and reduced bowel enhancement were the significant predictive factor for intestinal ischemia. There was no significant difference between the two groups regarding the duration of symptoms, peritoneal irritation sign, body temperature, CRP level, or the presence of mesenteric fluid.

### Development and validation of a scoring system for predicting intestinal ischemia

On the basis of the results of univariate analysis, clinical scores to predict intestinal ischemia requiring bowel resection were structured. The ischemia prediction score (IsPS) comprised 1 point each for WBC ≥ 10,000/L, BE ≤ -1.0 mmol/L, the presence ascites, and 2 points for reduced bowel enhancement (Table 3). The simple ischemia prediction score (s-IsPS) includes WBC, BE, and ascites. The modified ischemia prediction score (m-IsPS) includes WBC, BE, ascites and contrast-enhanced effect of intestinal wall evaluated by enhanced CT. Cut-off values of s-IsPS and m-IsPS were set based on ROC curve analysis (Figure S2). The optimal cut-off value was 2 for s-IsPS and 3 for m-IsPS.

**Table 3:** The ischemia prediction score (IsPS)

Variable	Score points
White blood cell ≥ 10,000/L (WBC)	1
Base excess ≤ -1.0 mmol/L (BE)	1
Ascites	1
Reduced bowel wall enhancement	2

[Simple IsPS (s-IsPS)] = WBC + BE + Ascites

[Modified IsPS (m-isPS)] = WBC + BE + Ascites + Reduced bowel wall enhancement

The predictive abilities of each factor and IsPS are shown in Table 4. Among each factor, the presence of reduced bowel enhancement was the most valuable predictive factor (sensitivity 70.3%, specificity 80.0%). The AUC of s-IsPS was 0.716 and m-IsPS was 0.838, and they were higher than the AUC of each factor.

**Table 4:** The predictive value of each factor and clinical score in development cohort

	**Sensitivity**	**Specificity**	**AUC**
White blood cell ≥ 10,000/L	58.3%	67.2%	0.628
Base excess ≤ -1.0 mmol/L	47.2%	75.0%	0.611
CT: Ascites	88.9%	32.8%	0.601
CT: Reduced contrast enhancement	70.3%	80.0%	0.752
s-IsPS ≥ 2	69.4%	65.4%	0.716
m-IsPS ≥ 3	86.7%	76.0%	0.838

*AUC* Area under curve, *CT* Computed tomography, *s-IsPS* Simple ischemia prediction score, *m-IsPS* Modified ischemia prediction score

The sensitivity, specificity, and AUC of s-IsPS were 80.0%, 82.3%, and 0.812, respectively in VC. The sensitivity, specificity, and AUC of m-IsPS were 80.0%, 88.2%, and 0.841, respectively in VC.

## Discussion

In this study, we examined the risk factors of intestinal ischemia in SSBO patients requiring emergency laparotomy and developed clinical scoring system to predict intestinal ischemia. In summary, univariate analysis showed that high WBC, low BE, ascites and reduced bowel enhancement were significantly associated with bowel resection. Furthermore, clinical scores comprising each predictive factor had higher accuracy to predict intestinal ischemia.

Previous studies have also reported the risk factors (WBC, BE, ascites and reduced bowel enhancement) identified in the present study. The lactate level, BE, or creatinine kinase might reflect anoxic damage of small bowel [10–12], and WBC count or procalcitonin level might reflect inflammatory response [13]. In reports on imaging, ascites was identified as an independent risk factor for bowel resection [5, 14]. Ascites can be clearly observed on nonenhanced CT and it is comparatively easy to interpret its presence. Among them, bloody ascites is often observed in SSBO, and it has been reported that the presence of red blood cells in the peritoneal fluid increases according to the degree of strangulation [15]. In the present study, bloody ascites was also found to be associated with small bowel resection [OR: 3.89 (1.47–10.9), *p* values 0.005), but the OR results were similar to those for ascites overall. Therefore, we used ascites (bloody and non-bloody) as the clinical utility variable. Meanwhile, reduced bowel enhancement was reported as the most specific diagnostic value to predict surgical ischemia in SSBO [16]. This CT finding was considered the result of blockage of the bowel wall arteriovenous circulation. The CT sign of small bowel wall thickness was also reported as valuable for diagnosing ischemia [17], but we did not use this sign considering its difficulty in their assessment and cut-off. Although the presence of peritoneal irritation sign is considered important in the diagnosis of small bowel obstruction [3, 4, 18], we did not observe a significant association between the peritoneal irritation sign and the need for bowel resection. That may be because determining the presence of the peritoneal irritation sign is relatively subjective, and the decision would vary among clinicians.

We developed a predictive scoring system that may be useful for the rapid and accurate diagnosis of intestinal ischemia in SSBO by combining these parameters. The s-IsPS showed similar prediction accuracy with an AUC of 0.716 compared to the previous discriminant formula with an AUC of 0.735 that included ascites, peritoneal irritation sign and lactate [14]. Thereafter, the m-IsPS was structured by including evaluation of contrasted CT with objective criteria, and the diagnostic accuracy was elevated to the AUC of 0.838. Several studies have also included the bowel wall enhancement in the clinical score and the AUC exceeded 0.80 [5, 19]. Although the bowel wall attenuation played a pivotal role in their clinical scores, its radiological sign was based on the assessment of an experienced radiologist, and thus it may be difficult for non-specialized clinicians to use them in actual clinical settings. Furthermore, the more the increase in their clinical parameter or total score, the more complicated is the calculation and use in clinical practice. The present score consists of 4 variables and the total scores range from 0 to 5, striking a good balance between accuracy and usability.

This study has its strength and clinical implications. First, since some patients with asthma, kidney injury, or hyperthyroidism are contraindicated to enhanced CT [20, 21], we separately developed the prediction score with and without enhanced CT. Simple predictive score without enhanced CT may be useful for clinicians to predict the probability of intestinal ischemia, whereas a contrasted CT examination is needed to allow a more accurate assessment of the severity of SSBO. Second, unlike the previous study, we assessed the radiological sign of reduced bowel enhancement with objective criteria, which enables a wide variety of clinicians to interpret bowel-wall attenuation on CT. Third, these models have been validated in the validation cohort, and they may apply to similar settings. This study had several limitations. First, potential biases exist because of the retrospective study design, and the study population was relatively small. Second, we did not include patients who received conservative treatment without emergency laparotomy.

In conclusion, white blood cell (WBC) count, base excess (BE), ascites and reduced bowel wall enhancement were the significant predictive factors for bowel resection in SSBO patients. The clinical score consisting of each parameter allowed early and accurate identification of intestinal ischemia in SSBO.

## Supplementary Information

**Additional file 1:** **Figure S1.** Receiver operating characteristic (ROC) for predicting small bowel resection with the CT value of ischemic and no-ischemic intestinal wall. **Figure S2.** Receive r operating characteristic (ROC).   

## Data Availability

The data that support the findings of this study are available on request from the corresponding author.

## References

[1] Foster NM, McGory ML, Zingmond DS, Ko CY (2006). Small bowel obstruction: a population-based appraisal. J Am Coll Surg.

[2] Fevang BT, Fevang J, Stangeland L, Soreide O, Svanes K, Viste A (2000). Complications and death after surgical treatment of small bowel obstruction: A 35-year institutional experience. Ann Surg.

[3] Balthazar EJ (1994). George W. Holmes Lecture.  CT of small-bowel obstruction. AJR Am J Roentgenol..

[4] Hayanga AJ, Bass-Wilkins K, Bulkley GB (2005). Current management of small-bowel obstruction. Adv Surg.

[5] Schwenter F, Poletti PA, Platon A, Perneger T, Morel P, Gervaz P (2010). Clinicoradiological score for predicting the risk of strangulated small bowel obstruction. Br J Surg.

[6] Choudhry AJ, Haddad NN, Rivera M, Morris DS, Zietlow SP, Schiller HJ (2016). Medical malpractice in the management of small bowel obstruction: A 33-year review of case law. Surgery.

[7] Ten Broek RPG, Krielen P, Di Saverio S, Coccolini F, Biffl WL, Ansaloni L (2018). Bologna guidelines for diagnosis and management of adhesive small bowel obstruction (ASBO): 2017 update of the evidence-based guidelines from the world society of emergency surgery ASBO working group. World J Emerg Surg.

[8] Frager D, Baer JW, Medwid SW, Rothpearl A, Bossart P (1996). Detection of intestinal ischemia in patients with acute small-bowel obstruction due to adhesions or hernia: efficacy of CT. AJR Am J Roentgenol.

[9] Ohira G, Shuto K, Kono T, Tohma T, Gunji H, Narushima K (2012). Utility of arterial phase of dynamic CT for detection of intestinal ischemia associated with strangulation ileus. World J Radiol.

[10] Takahashi R, Akagi Y, Tanaka T, Kaibara A, Kajiwara S, Shima I (2014). Clinicopathological evaluation of anoxic mucosal injury in strangulation ileus. BMC Surg.

[11] Tanaka K, Hanyu N, Iida T, Watanabe A, Kawano S, Usuba T (2012). Lactate levels in the detection of preoperative bowel strangulation. Am Surg.

[12] Markogiannakis H, Memos N, Messaris E, Dardamanis D, Larentzakis A, Papanikolaou D (2011). Predictive value of procalcitonin for bowel ischemia and necrosis in bowel obstruction. Surgery.

[13] Jancelewicz T, Vu LT, Shawo AE, Yeh B, Gasper WJ, Harris HW (2009). Predicting strangulated small bowel obstruction: an old problem revisited. J Gastrointest Surg.

[14] Ozawa M, Ishibe A, Suwa Y, Nakagawa K, Momiyama M, Watanabe J (2021). A novel discriminant formula for the prompt diagnosis of strangulated bowel obstruction. Surg Today.

[15] Kobayashi S, Matsuura K, Matsushima K, Okubo K, Henzan E, Maeshiro M (2007). Effectiveness of diagnostic paracentesis and ascites analysis for suspected strangulation obstruction. J Gastrointest Surg.

[16] Millet I, Taourel P, Ruyer A, Molinari N (2015). Value of CT findings to predict surgical ischemia in small bowel obstruction: A systematic review and meta-analysis. Eur Radiol..

[17] Millet I, Taourel P, Ruyer A, Molinari N (2015). Value of CT findings to predict surgical ischemia in small bowel obstruction: a systematic review and meta-analysis. Eur Radiol.

[18] Eskelinen M, Ikonen J, Lipponen P (1994). Contributions of history-taking, physical examination, and computer assistance to diagnosis of acute small-bowel obstruction. A prospective study of 1333 patients with acute abdominal pain. Scand J Gastroenterol..

[19] Bouassida M, Laamiri G, Zribi S, Slama H, Mroua B, Sassi S (2020). Predicting intestinal ischaemia in patients with adhesive small bowel obstruction: a simple score. World J Surg.

[20] Kodzwa R (2019). ACR Manual on Contrast Media: 2018 Updates. Radiol Technol.

[21] Tsushima Y, Ishiguchi T, Murakami T, Hayashi H, Hayakawa K, Fukuda K (2016). Safe use of iodinated and gadolinium-based contrast media in current practice in Japan: a questionnaire survey. Jpn J Radiol.

