# Supplementary material for: Ischemia prediction score (IsPS) in patients with strangulated small bowel obstruction: a retrospective cohort study
Source: BMC Gastroenterol. 2023 Apr 24;23:133. doi: 10.1186/s12876-023-02761-z (PMC10124058; doi:10.1186/s12876-023-02761-z)
Supplement: Supplementary file 1 — Additional file 1: Figure S1. Receiver operating characteristic (ROC) for predicting small bowel resection with the CT value of ischemic and no-ischemic intestinal wall. Figure S2. Receive r operating characteristic (ROC). [file 12876_2023_2761_MOESM1_ESM.docx]

**SUPPLEMENTARY MATERIALS**

**Figure S1. Receiver operating characteristic (ROC) for predicting small bowel resection with the CT value of ischemic and no-ischemic intestinal wall**

**Figure S2. Receive** **r operating characteristic (ROC)**

(A) **The simple ischemia prediction score (s-IsPS). (B) The modified ischemia prediction(m-IsPS)**
